# Comparison of Effects from Ultrasound Thawing, Vacuum Thawing and Microwave Thawing on the Quality Properties and Oxidation of Porcine *Longissimus Lumborum*

**DOI:** 10.3390/foods11091368

**Published:** 2022-05-09

**Authors:** Bo Wang, Xue Bai, Xin Du, Nan Pan, Shuo Shi, Xiufang Xia

**Affiliations:** College of Food Science, Northeast Agricultural University, Harbin 150030, China; wangbo9214@163.com (B.W.); snowbx1029@163.com (X.B.); dbnydxdx@163.com (X.D.); pannan36@163.com (N.P.); shishuo0902@163.com (S.S.)

**Keywords:** thawing method, porcine *longissimus lumborum*, texture, fluid losses, oxidation

## Abstract

The effects of vacuum thawing (VT), ultrasound thawing (UT) and microwave thawing (MT) on the quality, protein and lipid oxidation, internal temperature distribution and microstructure of porcine *longissimus lumborum* were compared. The results showed that a significant decrease (*p* < 0.05) in quality compared with those of fresh meat (FM) occurred for all of the thawing samples, especially for the MT samples. Changes in quality of the VT and UT samples were less significant than those of the MT samples. The increases in carbonyl content and TBARS value indicated that proteins and lipids in the thawing samples were oxidized. The decreases in uniform degrees of internal temperature distributions of muscles from the thawing samples were analysed by infrared thermography. Scanning electron microscopy images showed that the myofibril arrangements of thawing samples were looser than those of the FM samples with compact and ordered structure, which was proven by the obvious increase in the myofibril gap value of the thawing samples.

## 1. Introduction

The thawing processes represent an important step performed prior to the further processing of frozen meat, such as slicing, chopping and subsequent cooking [1]. Generally, the time of the thawing process is longer than that of freezing process, and long thawing times can potentially promote physicochemical changes. In the thawing process, some undesirable effects on muscle quality include those related to flavour [2], texture [3] and colour [4], while denaturation and aggregation [5,6] of protein can also occur through some physical and chemical changes.

The quality decrease in meat during thawing depends on many factors, such as thawing methods [7], thawing time [8] and thawing temperature [9]. Compared to common thawing methods (refrigerator [10], water-assisted [11] and air thawing [9]), some efficient and timesaving thawing methods, including high-voltage electrostatic field [12], vacuum [3], microwave [13] and ultrasonic thawing [14], have been widely applied to frozen foods such as fish, beef, edamame and mango. Cai et al. [5] noted that low temperature, fast thawing methods offer many advantages, such as shortened thawing time, reduced oxidation reaction and promoted quality.

Ultrasound has been verified to function effectively for frozen foods, saving thawing time and improving the qualities of the meat [15]. Previous studies have reported that the melting speed of ice crystals is faster than that of most common thawing methods, shortening the thawing times of samples [16]. For example, the thawing times of mango [8], edamame [17] and frozen fish [18] induced by ultrasound thawing were reduced by 51–73%, 54.39% and 71%, respectively. In addition, the insignificant negative impacts on pH and microbial growth in pork [19] and sensory properties of the blocks of Pacific cod [18] were compared between ultrasound thawing and common thawing. For vacuum thawing, the frozen samples were thawed in a low oxygen environment which exerts reduced effects on microbial reproduction and oxidation reactions [20]. The insignificant changes in gel qualities and protein structure induced by vacuum as well as vacuum-assisted thawing were observed compared with control group [3,20]. Microwave thawing can control bacterial growth and the quality reduction because of shorter thawing time [21]. Some studies suggested that the thawing rate of frozen pork thawed by microwave was 100 times faster than that of air thawing [22], and the quality of native starch sauces thawed by microwave was improved by inhibiting additional amylose degradation compared with water bath [23].

In our previous studies, the effects of freezing storage on the quality [24,25,26,27,28] and thawing methods on the structure [7] and gelling properties [1] of proteins have been evaluated. Most other research concerning thawing was related to the changes in muscle quality characters and oxidation induced by single thawing methods [19,29]. Nevertheless, less attention was received with respect to comparison of the effects between novel thawing methods and conventional thawing methods on pork quality traits. Therefore, in this experiment, the comparison of vacuum thawing (VT), ultrasound thawing (UT) and microwave thawing (MT) on the quality traits, protein and lipid oxidation, internal temperature distribution and microstructure of porcine *longissimus lumborum* was evaluated.

## 2. Materials and Methods

### 2.1. Materials

*Longissimus lumborum* (24 h post-mortem) purchased from local commercial abattoir (Harbin, China) were obtained from eight pork carcasses (total pork carcasses number in three independent batches) of similar age. All chemical reagents used were of analytical grade.

### 2.2. Sample Preparation

A total of 120 chop samples (100 ± 0.1 g, total samples number in each independent batches) were divided into four groups, and each group was subjected to the freeze process (−18 °C, 7 days). Thirty chop samples were randomly picked for each experimental groups and then thawing operation was performed. Due to the space limitation of thawing room, five chop samples were thawed each time. The frozen muscle chops were thawed using four different methods: VT, vacuum thawing (25 °C, 30 min), UT, ultrasonic thawing (20 °C, 20 min), MT, microwave thawing (5 min) and WT, water immersion thawing (14 °C, 55 min). To monitor the temperature changes, a temperature recorder (Applent Precision Instrument Co., Ltd., Changzhou, China) was inserted at the centre of the samples. The endpoint temperature of thawing was set to approximately 4 °C. VT was carried out in a vacuum chamber including a water tank (9 KPa). UT was carried out in an ultrasonic chamber (500 W, Nanjing Xianou Co., Ltd., Nanjing, China). MT was carried out in a microwave oven (800 W, BE525LMS0W, Siemens, 594 × 382 × 317 mm, Guangdong, China).

### 2.3. MP Extraction

The thawing muscles were chopped into small pieces for the myofibrillar protein (MP) extraction based on the method described by Li et al. [30] and Du et al. [31]. The measurement of protein concentration was carried out using bovine serum albumin as a standard.

### 2.4. Thawing Loss of Muscle

$$\mathrm{Thawing}\;\mathrm{loss}\;\left(\%\right)=\frac{W_{1}-W_{2}}{W_{1}}\times 100\%$$

where *W*_1_ represents the weight of porcine samples before thawing, and *W*_2_ represents the weight of porcine samples after thawing.

### 2.5. Cooking Loss of Muscle

The cooking loss from thawed samples was measured according to the methods described by Jin et al. [32] with slight modification. The thawed porcine chops were weighed (*W*_1_) and cooked in an 80 °C water bath until the centre temperature reached 75 °C. The samples were weighed (*W*_2_) after cooking. The cooking loss is defined as follows:

$$\mathrm{Cooking}\;\mathrm{loss}\;\left(\%\right)=\frac{W_{1}-W_{2}}{W_{1}}\times 100{\%}_{\;}$$

### 2.6. Colour of Muscle

The measurement of muscle colour was carried out based on the method described by Li et al. [33] with a colourimeter (ZE-6000, Illuminant D65; Nippon Denshoku, Tokyo, Japan). The thawed samples were balanced to room temperature and cut into slices with a diameter of 18 mm. The instrument was calibrated using a white standard plate (L* = 90.26, a* = −1.29, b* = 5.18). The values, expressed as L* (lightness), a* (redness), b* (yellowness) and Δ*E* units, were obtained from four different areas on the surface of each chop, and a minimum of 3 chops per treatment block was analysed to obtain an average value. The values of chroma (chroma = [a*^2^ × b*^2^] × 0.5) and hue angle (hue angle = arctan [b*/a*]) were calculated.

### 2.7. Texture Analysis of Muscle

The texture of muscle was analysed according to the determination of shear force, hardness, springiness, cohesiveness and chewiness of muscle. The muscle chops were cooled to room temperature (18 °C) after cooking.

The shear force was determined by using a tenderization analysis (C-LM3B tenderization instruments, Northeast Agricultural University, Harbin, China) with a 25 kg load transducer according to the research by Wang et al. [34] and Holman et al. [35] with slight modifications. The cooled muscle chops (18 °C) were cut into strips (1.5 × 1.5 × 5.0 cm) parallel to the muscle fibre orientation for the test, and a crosshead speed of 200 mm/min. The greatest force value was recorded as the shear force when the samples were cut.

The hardness, springiness, cohesiveness and chewiness of muscle was determined by Texture profile analysis (TPA) according to the method described by Pan et al. [36] at room temperature using a TA-XT plus Texture Analyser (Stable Micro System, Surrey, UK). The cooled muscle chops were cut into uniform cubic size (2 × 2 × 2 cm^3^) and subjected to two-cycle compression with a P/50 flat-surface cylindrical probe, 5 cm diameter cylindrical probe and 5 mm/s cross-head speed. Prior to analysis, samples were placed on the centre of the TPA platform and compressed to 40% of their original height. The conditions were as follows: pre-test speed 1.0 mm/s, test speed 1.0 mm/s, post-test speed 5.0 mm/s and the testing interval 5 s.

### 2.8. Moisture Mobility and Distribution of Muscle

The moisture mobility and distribution from thawed samples were evaluated using transverse relaxation time (T_2_). The low-field nuclear magnetic resonance (LF-NMR) relaxation time was measured according to the method of Jin et al. [37] with a LF-NMR analyser minispec mq 20 (Bruker Optik GmbH, Ettlingen, Germany) with a magnetic field strength of 0.47 T corresponding to a proton resonance frequency of 20 MHz. The thawed samples were cut into parallelepipeds (1 × 1 × 2 cm^3^) and placed in NMR tubes (18 mm of diameter). The transverse relaxation time (T_2_) was measured using the Carr–Purcell–Meiboom–Gill pulse sequence. For each sample, 16 scans were obtained at 2 s intervals with 3000 echoes in total. The continuous distribution of exponentials related to water located in different muscle compartments was fitted for all Carr–Purcell–Meiboom–Gill curves using the CONTIN algorithm after normalizing the raw data. Three relaxation times (T_2b_, T_21_ and T_22_) and their corresponding area fractions (P_2b_, P_21_ and P_22_) were recorded as outputs.

### 2.9. Oxidation Reaction

Protein oxidation was evaluated according to the content of carbonyl groups based on Jin et al. [37]. Carbonyl groups were detected by reaction with DNPH to form protein hydrazones. Myofibrillar protein solution was precipitated with 100 g/L TCA (*w*/*v*; final concentration). After centrifugation (2000× *g*, 10 min), pellet was treated with 2 g/L DNPH in 2 mol/L HCl with agitation for 1 h at room temperature. The fractions were then precipitated with 100 g/L TCA (final concentration) and centrifuged. The pellets were washed twice with 1 mL of ethanol:ethyl acetate (1:1 *v*/*v*), and the solution was precipitated with 100 g/L TCA (final concentration) and centrifuged. Proteins were then dissolved in 2 mL of 6 mol/L guanidine with 20 mmol/L sodium phosphate buffer at pH 6.5. Absorbance was measured at 365 nm for the DNPH-treated sample against an HCl control. The amount of carbonyl was expressed as nmol of DNPH fixed/mg of protein using an absorption coefficient of 22,000 (mol/L)^−1^ cm^−1^ for protein hydrazones.

Lipid oxidation was evaluated according to Wang and Xiong [38] with slight modifications. An accurately weighed finely chopped meat sample (ca. 0.4× *g*) was placed in a 25 mL screw cap test tube, and three drops of antioxidant solution (BHA), 3 mL of TBA solution and 17 mL of TCA-HCI solution were subsequently added. The mixture was vortexed, flushed with nitrogen gas and then heated in boiling water (100 °C) for 30 min. After being cooled to room temperature, a 5 mL aliquot of the suspension was mixed by vortexing with 5 mL of chloroform for 1 min, followed by centrifugation at 1800× *g* for 10 min. The upper phase (aqueous) was centrifuged again for 10 min under the same condition, and the absorbance of the supernatant was read at 532 nm with Ultraviolet spectrophotometer. The TBARS value, expressed as mg of malonaldehyde/kg of muscle sample, was calculated by using the following equation:

$$\mathrm{TBARS}\;\left(\mathrm{mg}/\mathrm{kg}\right)=\frac{A_{532}}{W_{s}}\times 9.48\;$$

here A_532_ is the absorbance (532 nm) of the assay solution, W_s_ is assigned to the weight of meat sample (g); ‘9.48’ corresponds to a derived constant.

### 2.10. Internal Temperature Distribution of Muscle

The measurement of internal temperature distribution of muscle was performed according to the procedure reported by Choi et al. [22] with a slight modification. The sample was immediately bisected vertically when the central temperature of thawed muscle reached approximately 4 °C, and the thermographic image of muscle was acquired by an infrared camera (GTC40, Bosch Co., Stuttgart, Germany) within 20 s.

### 2.11. Microstructure of Muscle

Images of microstructure from muscle were acquired by a scanning electron microscope (SEM) (S-3400N; Hitachi, Tokyo, Japan) according to the method reported by Wang et al. [39]. The samples (0.5 × 0.5 × 0.25 cm^3^, cut with a sharp-edged razor) were fixed with 25 mL/L glutaraldehyde in 0.2 mol/L phosphate buffer (pH 7.2) for 2 h. The samples were then rinsed for 1 h with distilled water before being dehydrated in ethanol with serial concentrations of 500, 700, 800, 900 and 1000 mL/L. Dried samples were mounted on a bronze stub and sputter-coated with gold (Sputter coater SPI-Module, West Chester, PA, USA). The microstructures of the samples were examined using a SEM at an operating voltage of 3.0 kV.

### 2.12. Sensory Evaluation and Consumer Testing

Once each experiment for a given thawing process was complete, 15 chop samples were randomly picked and cooked at 80 °C until the centre temperature reached 75 °C. The cooled muscle chops (18 °C) were cut into strips (1.0 × 1.0 × 2.0 cm^3^) parallel to the muscle fibre orientation for the sensory evaluation and consumer testing. Each chop samples could be divided into 18 strips to ensure that each sample could be tasted at least twice.

Panellists were asked to evaluate the appearance, tenderness, juiciness, flavour, overall acceptability of the cooked muscles. All panellists received samples in the same order with a 20-min rest period between samples. During the evaluation, the panellists were situated in a private booth under incandescent light. In order to clean the palate between the experiments, room temperature water was used. All samples were coded with three-digit random numbers and served in a random order to the panellists. Twenty panellists who had prior experience with meat product evaluation assessed the sensory attributes of cooked muscles using a 9-point scale. Scores were recorded on a scale of 1–9 (1 = extreme dislike, 9 = extreme like). Scores with means from 5 to 9 were considered acceptable. The mean scores from panellists for each sample and session were calculated and analysed.

Consumer testing: Consumer testing was analysed by the method of Heck et al. [40] and Ares and Jaeger [41]. One hundred and five consumers (46% male, 54% female, aged 18–55 years) were asked to complete a check-all-that-apply (CATA) questionnaire with four attributes defined by the trained panel as described above. The consumers were asked to check all the terms that they considered appropriate to describe each sample. Filtered water were provided to the consumers for mouth cleansing between samples. The sensory terms listed were randomized within and across consumers, meaning that each consumer received the CATA question with the terms in different order and this order was modified from sample to sample within the test.

Analysis of the CATA questionnaire: Correspondence Analysis (CA) was used to analyse data from the CATA questionnaire, considering the chi-square distance [42], calculated on the matrix containing the use frequency of attributes (Brightness, Mouthfeel, Juiciness, Pleasant) for each sample.

### 2.13. Statistical Analysis

All experiments were conducted three times (three independent batches), and each sample was evaluated in triplicate to evaluate the changes in quality properties and oxidation of pork from different thawing samples. The results are expressed as means values ± standard deviation (SD). One-way analysis of variance (ANOVA) was carried out followed by Duncan’s test using SPSS 22.0 (SPSS Inc., Chicago, IL, USA). The level of significance was set to *p* < 0.05. All graphs were generated using SigmaPlot 12.5.

## 3. Results and Discussion

### 3.1. Quality Traits

#### 3.1.1. Fluid Losses

Thawing loss is a vital quality indicator for frozen muscle because it would result in not only the change in weight but also decrease in quality, ultimately causing economic loss [43]. Table 1 showed that the thawing losses from different thawing samples increased obviously (*p* < 0.05). During the thawing process, the water melted by ice crystals in/outside the cell encountered difficulty in returning to the initial position in the fresh meat, which caused the decrease in the water-holding capacity [44]. The thawing loss of the MT samples was the highest (4.71%) among all thawing samples. Boonsumrej et al. [45] found that the high thawing loss of tiger shrimp was mainly induced by evaporated water upon instantaneous high temperature during microwave thawing. In addition, there was significantly low (*p* < 0.05) thawing loss of the samples treated with VT (2.8%) and UT (3.0%) compared with WT (3.7%). The low thawing loss primarily occurred on account of the low oxygen environment during VT, which slowed oxidation and decreased the degree of protein degeneration. Sun et al. [44] asserted that protein oxidation may result in degeneration and changes in the structure of protein, which caused the reduction in the water-binding ability of protein.

The cooking loss of FM was 18.70%, and there were increments of 10.7%, 3.6%, 23.4% and 27.1% after UT, VT, MT and WT, respectively (Table 1). Choi et al. [22] also showed the increased cooking loss of porcine *longissimus dorsi* after thawing was observed. The cooking loss from VT samples showed an insignificant increase (*p* > 0.05), and UT, MT and WT samples showed significant increases (*p* < 0.05) as compared with FM samples. The result in cooking loss could resulted in the decrease in water-holding capacity of muscle tissue and the degradation of myofibrillar protein, especially myosin [46]. Wang et al. [7] reported that the decrease in Ca^2+^-ATPase activity of MP from VT and UT samples was insignificant compared with that from FM (*p* > 0.05), while the significant decrease in that from MT samples was observed (*p* < 0.05), suggesting that the protein integrity of MT samples was severely destroyed and degraded. Combined with the results of thawing loss and cooking loss, VT and UT were more conducive to maintaining water-holding capacity of muscle than MT.

#### 3.1.2. Colour of Muscle

Colour is an important sensory attribute of meat because it determines the purchasing desire of consumers [47]. Table 2 shows the changes in colour of different thawing samples. Compared with FM samples, the a* value and chroma of different thawing samples decreased significantly (*p* < 0.05), while L*, Δ*E* and hue value increased significantly (*p* < 0.05).

The L* values can be used as a marker to judge the degree of muscle loss. The higher the muscle quality, the lower the L* values. Decreased water content may lead to increased reflection of light [48]. Table 2 showed that L* value from different thawing samples increased obviously (*p* < 0.05). The L* value from UT, VT, MT and WT samples were 2.44%, 1.20%, 8.07% and 10.07% higher than that from FM samples respectively (*p* < 0.05). Compared with UT and VT samples, significantly higher L* value from MT samples was obtained (*p* < 0.05). The difference in L* value among thawing samples may be related to the moisture content, state and distribution of thawing samples. Zhang et al. [49] demonstrated that samples with lower water content possessed higher L* values. The high thawing loss from MT samples led to stronger light reflection and lighter colour. The increase of L* value from UT samples may be attributed to the thawing environment. The UT samples were immersed in water during thawing, and part of water could be infiltrated into the poly nylon pouch packed with muscle, increasing the light reflection and L* value. The lower L* value from VT samples was not only due to the less thawing loss, but also due to the minimum moisture migration proved by LF-NMR analysis, which resulted in less thawed water attached to the meat surface and reduced the light reflection intensity [50].

The a* value from UT, VT, MT and WT samples decreased by 12.84%, 19.05%, 24.03% and 13.49% compared with that from FM samples, respectively (*p* < 0.05). Compared with VT and MT samples, a* value from UT samples decreased significantly (*p* < 0.05). The decrease in a* value during thawing was mainly due to the decrease in the amount of myoglobin, the main pigment in the meat, and the change in its chemical state [51]. The lower a* value from MT samples may be attributed to the loss of methemoglobin reducing enzyme with the exudation. The higher thawing loss from MT samples has been showed in Table 1. The metmyoglobin reducing enzyme is very active in fresh muscle and the metmyoglobin formed is quickly reduced to deoxymyoglobin and oxygenated back to oxymyoglobin, thereby retaining the colour. However, the metmyoglobin reducing enzyme could be lost from the sarcoplasmic environment as exudate during thawing, leading to the accumulate of metmyoglobin on the muscle surface and accelerating the loss of redness [52]. Muela et al. [53] also found that the decrease in redness of frozen muscle was related to the decrease in metmyoglobin reducing enzyme activity. In addition, the protein and lipid oxidation from MT samples caused by local overheating could increase the quantity of free radicals, leading to increased rates of myoglobin oxidation and metmyoglobin formation [54]. Choi et al. [22] reported that the a* value of pork loin from microwave thawing was lower than those from other thawing methods (radio frequency, water immersion and forced-air convection thawing). Although the less thawing loss and lower protein and lipid oxidation occurred, the low a* value from VT was obtained, which may be explained by the fact that myoglobin could be oxidized into metmyoglobin in a low oxygen partial pressure environment. In addition, the decrease in a* values from the UT samples resulted from the loss of water-soluble myoglobin with the increase in thawing loss during thawing [55].

The change in b* value from thawing samples was similar to that in L* value. The b* value from MT samples was higher than that from UT and VT samples (*p* < 0.05). Meanwhile, the change in b* value can also be demonstrated from the increase in hue angle from thawing samples. The hue angle was used to illustrate the colour change from red to yellow, and the increased hue angle indicated that the muscle subjected to thawing exhibited a more yellowish hue [56]. The higher b* value and hue angle from MT samples may be attributed to the high degree of protein oxidation. Coombs et al. [57] revealed that protein oxidation could increase b* values and hue angle in the process of freezing and thawing. This result could be related to the increase in TBARS during thawing. However, the hue angle from VT samples was lower than that form UT samples, which may be due to the low a* value induced by low oxygen partial pressure environment.

Chroma value represents colour intensity and has been considered a good indicator of meat stability [58]. In this study, the chroma value from thawing samples decreased significantly (*p* < 0.05), suggesting that thawing process was not conductive to maintaining the stability of muscle colour [59]. The chroma values from VT and MT samples were significantly lower than those from UT samples (*p* < 0.05), which illustrated that VT and MT were not conducive to maintaining the stability of muscle colour.

#### 3.1.3. Texture of Muscle

The shear force and texture (hardness, cohesiveness, chewiness and springiness) was used to analyse the texture of muscle. The measurement of shear force from muscle is carried out to assess tenderness. The higher tenderness in muscle, the lower shear force [60]. As shown in Table 3, the shear force value from VT, UT, MT and WT samples was obviously increased by 10.8%, 17.8%, 36.9 and 34.5%, respectively, compared with FM (28.89 N) (*p* < 0.05), which illustrated that the tenderness of different thawing samples decreased. The decrease in tenderness from frozen and thawed samples may be caused by the high thawing and cooking loss [25] and the shrinkage and cracking of muscle fibres [61]. The results of thawing and cooking loss during thawing have been demonstrated in Table 1. Lagersted et al. [62] reported that decreased tenderness value was due to the loss of fluid during thawing, which led to the decrease of water available for hydration of muscle fibres. Furthermore, toughening is caused by sarcomere shortening in the process of thawing. Dransfield et al. [63] found that the muscle length of beef thawed at room temperature was shortened by roughly 40% and endowed the meat with greater hardness. Lagerstedt et al. [62] also reported significantly reduced tenderness from thawed beef meat.

There was no significant difference in shear force value between VT and UT samples (*p* > 0.05). However, the shear force value from MT samples was significantly lower than that from VT and UT samples (*p* < 0.05). The decreased tenderness during thawing may also be due to structural changes in proteins induced by oxidation [45]. The low tenderness of muscle treated with MT may be linked to protein oxidation of porcine *longissimus dorsi* induced by instantaneous high temperature during thawing. Xia et al. [25] verified that protein oxidation of pork during MT process might be due to the release of pro-oxidant and oxidative enzymes from ruptured cellular organs. However, protein oxidation and intra/inter-molecular cross-linking were inhibited during VT process because of the low oxygen environment, which contributed to maintain muscle tenderness [64].

The high tenderness of muscle treated with UT might be attributed to the quick thawing rate [7] and the high physical forces associated with shear force and turbulence from cavitation [7]. Thawing rate is one of the most important factors during thawing, because it controls the time at which proteins are oxidized [65]. A quick thawing rate during UT was due to the quick phase transition speed from ice to water in frozen samples. Ultrasound treatment could convert acoustic energy into heat energy by the collapse of cavitation bubbles causing a temperature rise in the frozen samples [66]. Jayasooriya et al. [67] found that during the ultrasound process, a large number of lysosomes were released into the sarcoplasm due to the shear force and turbulence caused by cavitation, thus accelerating the enzymatic hydrolysis reaction and increasing the muscle tenderness. Dickens et al. [68] also reported the tenderization effect of ultrasonic treatment on beef and poultry muscle. Overall, the negative effects of VT and UT on tenderness were lower than those of MT.

The measurement of texture is a direct and important method to assess the meat quality, which condition its palatability for consumers. Table 3 shows the changes in texture (hardness, cohesiveness, chewiness and springiness) from different thawing samples. Compared with FM samples, increased hardness value and decreased cohesiveness, chewiness and springiness values of muscle treated with different thawing methods were observed. It is noted that insignificant difference (*p* > 0.05) in hardness value from VT samples as well as cohesiveness and chewiness values from UT and VT samples were observed compared with the FM samples. Nevertheless, significantly higher (*p* < 0.05) hardness value and significantly lower (*p* < 0.05) cohesiveness, chewiness and springiness values were obtained in WT and MT samples with respect to other thawing samples. Mousakhani-Ganjeh et al. [69] also pointed out that the hardness value was increased, and the cohesiveness and springiness values were decreased during the thawing process, which might be attributed to the protein denaturation occurring in the proteins of fish during thawing.

### 3.2. Moisture Mobility and Distribution of Muscle

**Figure 1:**
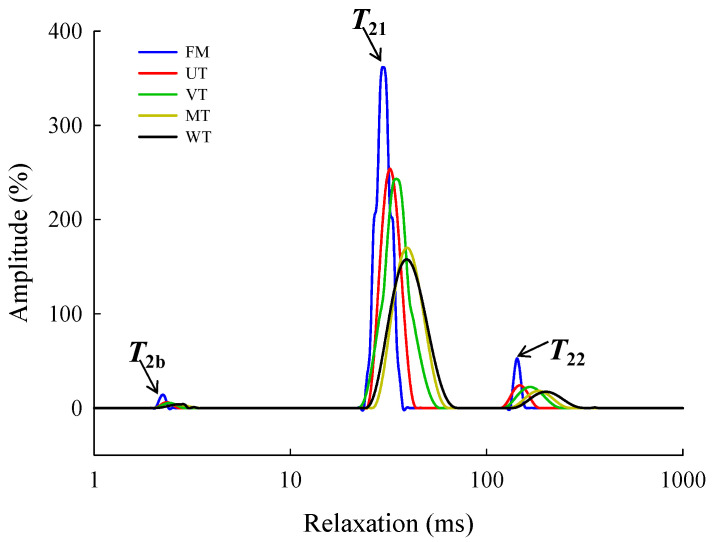
Change in distribution of the LF-NMR relaxation times of porcine *longissimus dorsi* induced by thawing methods. FM, fresh meat; UT, ultrasonic thawing (20 °C); VT, vacuum thawing (25 °C); MT, microwave thawing; WT, water immersion thawing (14 °C).

The mobility and distribution of three kinds of moisture in porcine *longissimus lumborum* could be analysed by the measurement of *T*_2_ (*T*_2b_, *T*_21_ and *T*_22_) and corresponding percentages (*P*_2b_, *P*_21_ and *P*_22_) of peak areas based on LF-NMR [44]. As shown in Figure 1, three peaks (*T*_2b_, *T*_21_ and *T*_22_) were showed and represented three distinct water populations. Table 4 shows the changes in *T*_2b_, *T*_21_ and *T*_22_ relaxation times of different thawing muscle. Insignificant difference (*p* > 0.05) in *T*_2b_ relaxation times of thawing samples was observed because bound water was very resistant to freezing or thawing [70]. Zhang et al. [71] also found that the change in *T*_2b_ relaxation time of porcine samples after thawing was not obvious compared with fresh muscle (*p* > 0.05), which was primarily attributed to the fact that bound water is not affected by any change in mechanical stress and microstructure of muscle. The significant increase (*p* < 0.05) in *T*_21_ relaxation times of thawing samples was observed. The prolonged relaxation times of water reflected the decrease in combination ability between water and protein and the increase in mobility of water [72]. It was worth noting that *T*_21_ relaxation times from the MT samples were significantly longer (*p* < 0.05) compared with other thawing samples, which signified that the tightness between immobilized water and protein molecules was weakened and that the mobility of immobilized water from MT was high. For *T*_22_, the changes in relaxation time from thawing samples were similar to those of *T*_21_. This result indicated that the free water of thawing samples encountered difficulty returning to the position as in the fresh meat because of the high mobility of free water in the destructed myofibrils.

Table 4 shows the percentages of peak areas (*P*_2b_, *P*_21_ and *P*_22_) corresponding to *T*_2_ relaxation time. Compared with the *P*_21_ values from FM samples (90.55%), the values of the VT, UT, MT and WT samples decreased by 4.61%, 4.10%, 8.71% and 6.70%, and the significant difference among thawing samples and the FM samples was observed (*p* < 0.05). The changing trend of *P*_21_ values from different thawing samples was consistent with the result that the WHC of porcine *longissimus lumborum* was decreased (Table 1). *P*_22_ values of the UT, VT, MT and WT samples increased by 44.23%, 39.26%, 83.49% and 64.13% compared with that of the FM samples (9.97%), respectively. The changes in *P*_21_ and *P*_22_ indicated that the immobilized water entrapped within the space between the thick and thin filaments or in the myofibrillar network was transformed into free water existed in the intercellular space during the thawing process. The result was consistent with the study of Han et al. [73], who reported that immobilized water was redistributed into the space between fibres, which led to the decline in the water-holding capacity of samples after the thawing process.

### 3.3. Protein and Lipid Oxidation

Protein and lipid oxidation were monitored by carbonyl content and TBARS, respectively (Table 5). The carbonyl content and TBARS from the VT, UT, MT and WT samples were increased by 2.9%, 3.8%, 8.6% and 5.7%, and 13.3%, 6.7%, 53.3% and 20.0% compared with FM samples, respectively. Kim et al. [74] confirmed that the post mortem processing of muscles, such as frozen storage and freezing–thawing cycles, could accelerate protein and lipid oxidation. The increases in carbonyl content and TBARS of muscle during processing were associated with the release of pro-oxidative factors involved with free radicals and oxidative enzymes [2]. Boonsumrej et al. [45] also pointed out that the disruption of muscle ultrastructure during freezing and thawing could release haem iron, oxidative enzymes and other pro-oxidants which accelerate the oxidation of muscle.

The differences in oxidation induced by UT and VT were not significant (*p* > 0.05), and their oxidation degrees were lower than that of MT. Xia et al. [25] reported that the TBARS of porcine longissimus muscle during microwave thawing was higher than during refrigerator thawing, which could be explained by the fact that the instantaneous high temperature induced by microwave thawing triggered the protein and lipid oxidation of muscle. The low carbonyl content and TBARS of samples might be attributed to the low speed of oxidation under the low oxygen environment during VT. Oxidation happening in the thawing process may result in the decrease in eating quality referred to with respect to tenderness, WHC and colour (Table 1).

### 3.4. Internal Temperature Distribution

**Figure 2:**
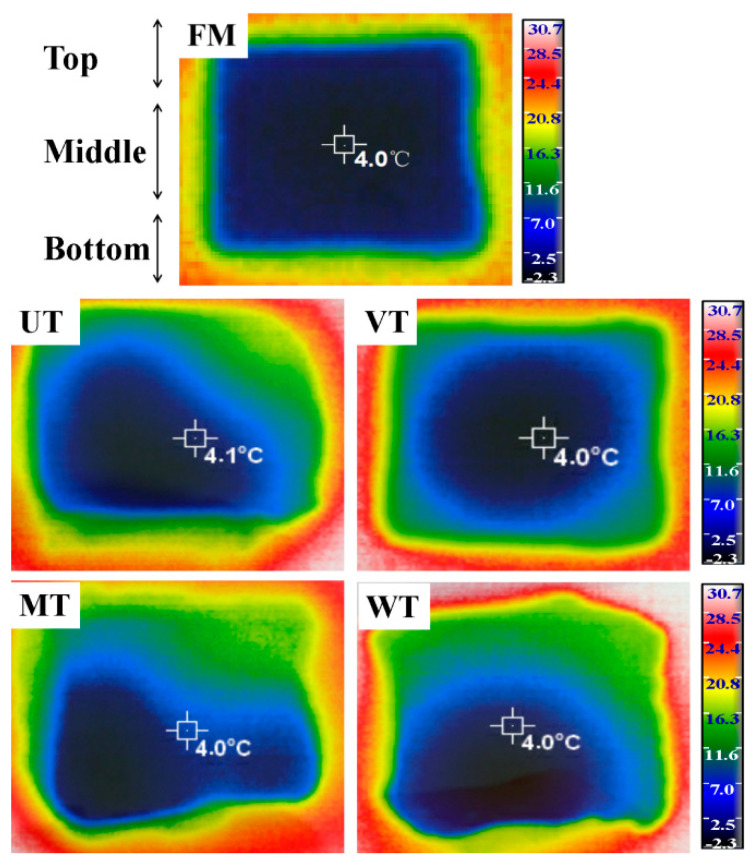
Change in internal temperature distribution of porcine *longissimus dorsi* induced by thawing methods. FM, fresh meat; UT, ultrasonic thawing (20 °C); VT, vacuum thawing (25 °C); MT, microwave thawing; WT, water immersion thawing (14 °C).

The changes in internal temperature distribution of muscle treated with different thawing methods were observed by thermographic imaging using infrared thermography (Figure 2). The coloration from blue (−2 °C, lower temperatures) to red (30 °C, higher temperatures) indicates the temperature change of muscle during thawing.

A clear difference in colour distribution of muscle between FM and thawing samples was observed. The colour distribution from FM samples was symmetric, which showed the uniform internal temperature distribution within muscle. The internal temperature distribution from VT samples during thawing exhibited the form of concentric circles transferring inwards from high to low temperature, which reflected the uniformity of thawing: at all points equidistant from the middle of the sample (the coldest point, 4 °C), the heat penetration is equivalent. The thawing patterns of muscle from the UT, MT and WT samples did not exhibit the concentric circle shape, which illustrated non-homogenous thawing patterns during UT, MT and WT processes. The inhomogeneous internal temperature distribution within muscle was mainly attributed to the local overheating phenomenon during UT and MT processes. This finding was consistent with previous studies representing local overheating at the edge region of shrimp [45] and beef [75] induced by microwave thawing. Li and Sun [76] also pointed out that some disadvantages such as localized heating could occur during ultrasound thawing. For the WT process, the non-uniform internal temperature distribution could be attributed to the low heat conductivity and heat diffusivity, which led to higher temperatures at the top than within the centre of the samples centre [22].

### 3.5. Microstructure of Muscle

**Figure 3:**
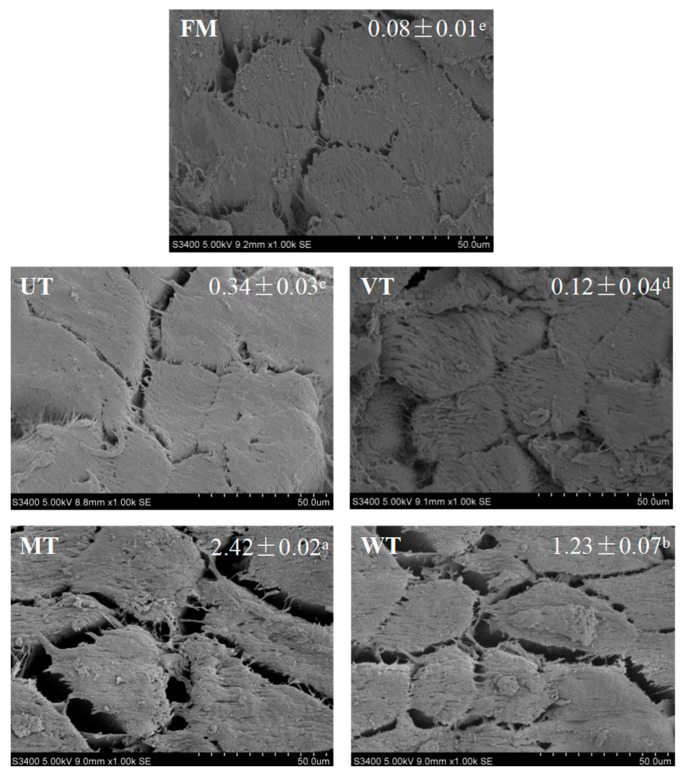
Change in microstructure of porcine *longissimus dorsi* induced by thawing methods. FM, fresh meat; UT, ultrasonic thawing (20 °C); VT, vacuum thawing (25 °C); MT, microwave thawing; WT, water immersion thawing (14 °C). The gap areas (mm^2^) are given as the means ± SD, with different lowercase letters (a–e) indicating significant differences (*p* < 0.05).

The changes in microstructure of different thawing samples are displayed in Figure 3. The intact and tight microstructure of the FM samples was destroyed, and the intermuscular gap was increased significantly (*p* < 0.05) after thawing. The intermuscular space of the VT samples (0.12 mm^2^) was smaller than for other thawing methods, which illustrated the destruction of VT to the microstructure of samples. The myofibre of the MT samples was separated, and the intermuscular gap was large compared with other thawing methods, which indicated that the negative effect of MT on the microstructure of samples was the greatest. The results were consistent with the study of Xia et al. [25], who reported that the destruction from MT samples on the microstructure was greatest among all thawing methods (refrigerator, ambient temperature, water immersion and lotic water thawing). Cai et al. [13] also suggested that the obvious shrinkage of muscle was induced by the overheating phenomenon during microwave thawing.

The increase in intermuscular gap during thawing may be attributed to the excessive dehydration of muscle induced by protein denaturation and the breakage of epimysium, perimysium and endomysium [77]. Xia et al. [25] also reported that protein denaturation and cell disruption could be induced by improper thawing processes. Choi [22] reported that the looser structure of muscle fibre was associated with low water-holding capacity and tenderness of pork loin during freezing and thawing. The obtained results of low water-holding capacity and high shear force were coincidental with the disrupted microstructure (Table 1).

### 3.6. Sensory Evaluation and Consumer Testing

The sensory properties of food products play a decisive role on the food choices of consumers. Table 6 shows the sensory attributes from different thawing samples. As shown in Table 6, differences among different thawing samples were found for tenderness, juiciness and overall acceptability (*p* < 0.05), while no difference were registered for appearance and flavour (*p* > 0.05). There was no significant difference in the appearance from different thawing samples, which may be due to the fact that the colour differences among cooked thawing samples were not easy to identify.

The significantly lower score of tenderness from MT samples was obtained (*p* < 0.05). Lagerstedt et al. [62] showed that a trained sensory panel rated the freeze/thawed meat significantly less tender than the chilled meat. This sensory result was attributed to the loss of fluid during thawing that resulted in less water available to hydrate the muscle fibres; thus, a greater quantity of fibres per surface area seemed to increase the toughness as perceived by the sensory panel [78]. The high thawing and cooking loss and protein oxidation from MT samples induced by instantaneous high temperature during thawing led to the low tenderness scores. However, protein oxidation was inhibited during the VT process because of the low oxygen environment, which contributed to maintaining muscle tenderness [64]. Meanwhile, the higher tenderness scores from UT samples could be linked to the quick thawing rate and high physical force associated with shear force and turbulence from cavitation [7]. The variation trend of juiciness scores from thawing samples was consistent with tenderness. The sensation of juiciness upon the mastication of a red meat product is a function of the water content of the meat. The increased juiciness in UT and VT samples could be linked with the quick thawing rate and low degree of oxidation, which could enhance water holding capacity. Meanwhile, the results that UT and VT samples were perceived by the tasters as more tender and juicier than MT samples influenced the overall acceptability.

Table 7 shows the results of the consumer preference test. CATA was applied using four sensory descriptors. The results of brightness, mouthfeel and juiciness from thawing samples were similar to that of sensory attributes. The brightness from thawing samples was also not easily identified. A bad mouthfeel from MT samples was obtained. Consumer satisfaction with the tenderness of a red meat product is based on the interaction between the textural properties of the meat and ‘mouthfeel’—this being an experience that includes mastication and biting properties (e.g., chewiness, hardness, firmness and softness). The texture results from MT samples showed higher hardness and lower cohesiveness, chewiness and springiness. The results of mouthfeel and juiciness from UT and VT samples led to the fact that more muscles were marked as ‘pleasant’ which indicated that the muscles were accepted. However, fewer muscles were marked as ‘pleasant’ which indicated that the muscles were less accepted.

## 4. Conclusions

The effects of thawing methods (UT, VT, MT) on quality properties of porcine *longissimus lumborum* were confirmed by the increases in thawing loss, cooking loss, shear force, L*, b*, Δ*E*, hue, hardness, relaxation time, the ratios of free water (*P*_22_), carbonyl contents and TBARS and the decreases in a*, chroma, cohesiveness, chewiness, springiness and the ratios of immobilized water (*P*_21_). The internal temperature distribution of muscle samples after thawing was nonuniform, except for in the case of VT. Based on the observation of SEM, the tight and intact myofibril structure of muscle was destroyed during thawing. The effects of MT on the quality properties of samples were high, and the sensory evaluation and consumer testing showed that the MT samples were less accepted. The UT and VT could maintain the quality better. However, the low a* value from VT samples caused by low oxygen environment and the inhomogeneous temperature distribution within muscle from UT samples caused by local overheating could not be neglected, which needs to be solved in further research and practical application.

## Figures and Tables

**Table 1:** Change in fluid losses of porcine *longissimus dorsi* induced by thawing methods.

Thawing Methods	Fluid Losses (%)	
	Thawing Loss	Cooking Loss
FM	-	18.70 ± 0.33 ^c^
UT	2.98 ± 0.29 ^c^	20.71 ± 0.72 ^b^
VT	2.78 ± 0.27 ^c^	19.37 ± 0.45 ^c^
MT	4.71 ± 0.15 ^a^	23.07 ± 0.24 ^a^
WT	3.70 ± 0.23 ^b^	23.77 ± 0.57 ^a^

The means in the same column with different lowercase letters (a–cdiffer significantly (*p* < 0.05). The results are mean ± SD (n = 3 × 3). FM, fresh meat; UT, ultrasonic thawing (20 °C); VT, vacuum thawing (25 °C); MT, microwave thawing; WT, water immersion thawing (14 °C).

**Table 2:** Change in colour of porcine *longissimus dorsi* induced by thawing methods.

Thawing Methods	L*	a*	b*	Δ*E*	Chroma	Hue (°)
FM	36.44 ± 0.12 ^e^	13.86 ± 0.23 ^a^	8.88 ± 0.06 ^d^	-	16.46 ± 0.43 ^a^	32.65 ± 0.16 ^d^
UT	37.33 ± 0.16 ^c^	12.08 ± 0.35 ^b^	9.08 ± 0.42 ^d^	2.00 ± 0.04 ^d^	15.11 ± 0.21 ^bc^	36.93 ± 0.25 ^c^
VT	36.88 ± 0.07 ^d^	11.22 ± 0.31 ^c^	9.17 ± 0.32 ^c^	2.69 ± 0.01 ^c^	14.49 ± 0.46 ^c^	39.26 ± 0.44 ^b^
MT	39.38 ± 0.14 ^b^	10.53 ± 0.19 ^c^	10.55 ± 0.32 ^a^	4.75 ± 0.06 ^a^	14.90 ± 0.24 ^c^	45.05 ± 0.26 ^a^
WT	40.11 ± 0.19 ^a^	11.99 ± 0.24 ^b^	10.01 ± 0.18 ^b^	4.27 ± 0.03 ^b^	15.62 ± 0.31 ^b^	39.85 ± 0.31 ^b^

The means in the same column with different lowercase letters (a–e) differ significantly (*p* < 0.05). FM, fresh meat; UT, ultrasonic thawing (20 °C); VT, vacuum thawing (25 °C); MT, microwave thawing; WT, water immersion thawing (14 °C).

**Table 3:** Change in shear force and texture (hardness, cohesiveness, chewiness and springiness) of porcine *longissimus dorsi* induced by thawing methods.

Thawing Methods	Shear Force (N)	Hardness	Cohesiveness	Chewiness	Springiness
FM	28.89 ± 0.49 ^c^	34.57 ± 0.52 ^d^	0.54 ± 0.01 ^a^	22.37 ± 0.43 ^a^	1.71 ± 0.02 ^a^
UT	34.03 ± 0.78 ^b^	37.09 ± 0.98 ^bc^	0.51 ± 0.01 ^ab^	21.07 ± 0.76 ^a^	1.56 ± 0.03 ^b^
VT	32.02 ± 0.84 ^b^	35.77 ± 0.27 ^cd^	0.52 ± 0.01 ^a^	21.45 ± 0.49 ^a^	1.60 ± 0.01 ^b^
MT	39.54 ± 0.98 ^a^	39.61 ± 0.54 ^a^	0.46 ± 0.02 ^c^	18.42 ± 0.41 ^b^	1.35 ± 0.03 ^d^
WT	38.86 ± 0.31 ^a^	38.39 ± 0.54 ^ab^	0.48 ± 0.01 ^bc^	19.43 ± 0.46 ^b^	1.42 ± 0.02 ^c^

Means in shear force, hardness, cohesiveness, chewiness and springiness with different lowercase letters (a–d) differ significantly (*p* < 0.05). FM, fresh meat; UT, ultrasonic thawing (20 °C); VT, vacuum thawing (25 °C); MT, microwave thawing; WT, water immersion thawing (14 °C).

**Table 4:** Change in distribution of the *T*_2_ relaxation time and the *P*_2_ (*T*_2_ peak ratio) of porcine *longissimus dorsi* induced by thawing methods.

Thawing Methods	*T*_2_ (ms)			*P*_2_ (%)		
	*T*_2b_	*T*_21_	*T*_22_	*P*_2b_	*P*_21_	*P*_22_
FM	1.52 ± 0.04 ^a^	26.23 ± 0.31 ^a^	114.33 ± 2.52 ^c^	0.14 ± 0.01 ^a^	90.55 ± 0.59 ^a^	9.45 ± 0.96 ^c^
UT	1.61 ± 0.02 ^a^	43.30 ± 0.40 ^c^	118.33 ± 2.08 ^cd^	0.11 ± 0.01 ^ab^	86.37 ± 1.08 ^b^	13.63 ± 1.76 ^b^
VT	1.57 ± 0.04 ^a^	37.43 ± 0.31 ^d^	116.67 ± 2.08 ^cd^	0.11 ± 0.02 ^ab^	86.84 ± 0.53 ^b^	13.16 ± 1.18 ^b^
MT	1.73 ± 0.02 ^a^	49.30 ± 0.56 ^a^	137.33 ± 3.21 ^a^	0.08 ± 0.01 ^b^	82.66 ± 0.68 ^c^	17.34 ± 1.73 ^a^
WT	1.62 ± 0.02 ^a^	45.53 ± 0.40 ^b^	126.67 ± 2.08 ^b^	0.09 ± 0.02 ^b^	84.49 ± 0.98 ^c^	15.51 ± 0.51 ^ab^

The means in the same *T*_2_ with different lowercase letters (a–d) differ significantly (*p* < 0.05). The means in the same *P*_2_ with different lowercase letters (a–d) differ significantly (*p* < 0.05). FM, fresh meat; UT, ultrasonic thawing (20 °C); VT, vacuum thawing (25 °C); MT, microwave thawing; WT, water immersion thawing (14 °C).

**Table 5:** Change in carbonyl content and TBARS of porcine *longissimus dorsi* induced by thawing methods.

Thawing Methods	Carbonyl Content nmol/mg MP	TBARS mg/kg MP
FM	1.05 ± 0.02 ^c^	0.15 ± 0.01 ^c^
UT	1.09 ± 0.02 ^b^	0.18 ± 0.01 ^b^
VT	1.09 ± 0.01 ^b^	0.16 ± 0.01 ^bc^
MT	1.14 ± 0.01 ^a^	0.23 ± 0.02 ^a^
WT	1.11 ± 0.02 ^ab^	0.18 ± 0.01 ^b^

FM, fresh meat; UT, ultrasonic thawing (20 °C); VT, vacuum thawing (25 °C); MT, microwave thawing; WT, water immersion thawing (14 °C). Means in carbonyl content with different lowercase letters (a,b) differ significantly (*p* < 0.05); Means in TBARS with different lowercase letters (a–c) differ significantly (*p* < 0.05).

**Table 6:** Sensory characteristics of cooked porcine *longissimus dorsi* induced by thawing methods.

Thawing Methods	Sensory Attributes				
	Appearance	Tenderness	Juiciness	Flavor	Overall Acceptability
FM	7.67 ± 0.25 ^a^	8.02 ± 0.17 ^a^	7.06 ± 0.13 ^a^	6.83 ± 0.15 ^a^	7.26 ± 0.25 ^a^
UT	7.52 ± 0.27 ^a^	7.79 ± 0.35 ^a^	6.69 ± 0.15 ^a^	6.77 ± 0.12 ^a^	7.04 ± 0.11 ^a^
VT	7.57 ± 0.16 ^a^	7.93 ± 0.24 ^a^	6.78 ± 0.11 ^a^	6.81 ± 0.26 ^a^	7.12 ± 0.14 ^a^
MT	7.38 ± 0.22 ^a^	7.04 ± 0.19 ^b^	6.15 ± 0.22 ^b^	6.58 ± 0.13 ^a^	6.33 ± 0.25 ^b^
WT	7.47 ± 0.21 ^a^	7.13 ± 0.27 ^b^	6.22 ± 0.10 ^b^	6.61 ± 0.33 ^a^	6.51 ± 0.11 ^b^

The means in the same column with different lowercase letters (a,b) differ significantly (*p* < 0.05). FM, fresh meat; UT, ultrasonic thawing (20 °C); VT, vacuum thawing (25 °C); MT, microwave thawing; WT, water immersion thawing (14 °C).

**Table 7:** Frequency mentioned attributes by porcine *longissimus dorsi* CATA for each sample.

Thawing Methods	Attributes			
	Brightness	Mouthfeel	Juiciness	Pleasant
FM	25	45	51	32
UT	23	43	47	28
VT	20	44	49	28
MT	20	37	40	23
WT	22	39	38	25

FM, fresh meat; UT, ultrasonic thawing (20 °C); VT, vacuum thawing (25 °C); MT, microwave thawing; WT, water immersion thawing (14 °C).

## Data Availability

The data presented in this study are available in the article.
